# A New Twist on an Animal Model Reveals Environmental Constraints on Selection

**DOI:** 10.1371/journal.pbio.0040236

**Published:** 2006-06-13

**Authors:** Liza Gross

No one knows for sure when or how they got there, but Soay sheep *(Ovis aries)* arrived on the isolated, wind-swept Scottish island of Soay sometime during the Bronze Age, thousands of years ago. In the 1930s, a flock was relocated to Hirta, an adjacent island in the St. Kilda archipelago, after villagers evacuated to the mainland. Since then, the sheep have been left mostly to their own devices, save for ongoing encounters with scientists keen to test a range of evolutionary hypotheses on this unique population.

Mark-and-recapture studies have revealed important clues to the ecology of the population, which periodically crashes, typically when a severe winter follows a robust reproductive season. These studies have also generated a wealth of information on birth and death rates, reproduction, pedigree (maternal and paternal lineages), and phenotype (such as birth weight).

Alastair Wilson, Loeske Kruuk, and colleagues took advantage of a 20-year study of birth weight in Soay sheep—which covered nine generations and significant climatic fluctuations—to examine the impact of environmental quality on the strength of selection and the amount of genetic variation associated with a fitness-related trait. Evolution should occur when selection acts on a trait, such as birth weight, that has a genetic basis. Birth weight is closely linked to juvenile mortality, with larger lambs having higher survival and higher fitness. The first six months of life are critical; lamb deaths during this crucial window account for nearly a quarter of all deaths.

Even though birth weight has all the raw materials for evolution—a positive correlation between birth weight and survival, and a heritable basis for variation, attributed to maternal genetic effects—previous studies found no evidence for evolution of the trait. Since annual birth weight had not increased over the 20 years, the researchers hypothesized that a variable environment could limit its micro-evolution by limiting heritability, selection, or both. Wilson et al. found evidence that that this is indeed the case.

To study the micro-evolutionary dynamics of a wild population, they adapted a theoretical model long used by animal breeders. Quantitative genetic models measure the genetic contribution (which typically involves multiple genes) to variation in a given trait, such as milk production in dairy cows. With these models, plant and animal breeders maintain or develop desired traits by predicting an evolutionary path based on the strength of selection and the amount of genetic variance underlying the trait. Some of these models assume a constant environment, which is fine for breeders, who can artificially control their subjects' environment, but proves problematic for evolutionary studies of wild populations.

To overcome these limitations, the researchers incorporated a statistical tool called random regression analysis. With this approach, they could not only model the effects of environmental variation on selection and heritability but also maximize the statistical power of their dataset. This is a particularly effective technique for natural populations, for which the data are inevitably limited. It also allowed the researchers to model the genetic variance underlying lamb birth weight as a function of environmental quality (poor if lamb survival was low, good if survival was high), creating a more realistic evolutionary picture. (Lamb birth weight is primarily influenced by “maternal genetic effects,” which derive from gene products of the mother's genome, rather than those of the lambs themselves.)

The most powerful modeling result showed that the extent to which birth weight is heritable—that is, the variance caused by maternal genetic effects—increased along with environmental quality. But when Wilson et al. modeled the relationship between birth weight and fitness (defined as lamb survival) across environments, they found that the strength of selection followed a different pattern. Fitness increased with birth weight, and with environmental quality, but the positive relationship between fitness and birth weight—indicating the strength of selection—became weaker in better environments.

Environmental quality shapes the trajectories for both genetic variance and the strength of selection. Lambs are not growing bigger and bigger because there's a lack of heritable variation for selection to act on in harsh environments and there's a lack of selection to act on higher genetic variation during favorable conditions. These results emphasize the importance of using biologically realistic models to predict the evolution of traits in wild populations—an important component of genetic restoration for endangered species, for example. They also confirm the value of long-term studies on wild populations to better detect patterns of selection and genetic variation in the natural world.

## 

**Figure:**
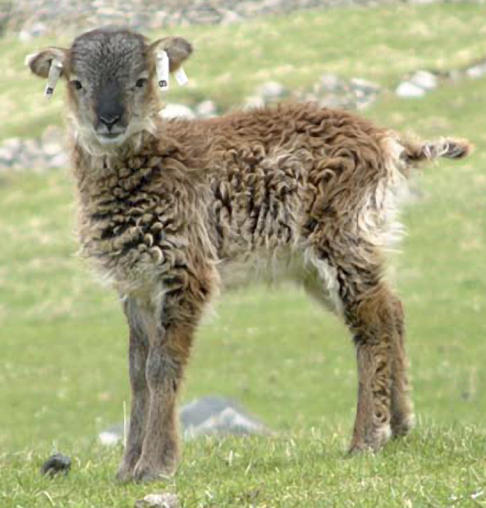
A Soay sheep lamb on the Scottish island of Hirta.

